# MDM2, MDM4 and EGFR Amplifications and Hyperprogression in Metastatic Acral and Mucosal Melanoma

**DOI:** 10.3390/cancers12030540

**Published:** 2020-02-26

**Authors:** Andrea Forschner, Franz-Joachim Hilke, Irina Bonzheim, Axel Gschwind, German Demidov, Teresa Amaral, Stephan Ossowski, Olaf Riess, Christopher Schroeder, Peter Martus, Bernhard Klumpp, Irene Gonzalez-Menendez, Claus Garbe, Heike Niessner, Tobias Sinnberg

**Affiliations:** 1 Center for Dermatooncology, Department of Dermatology, University Hospital Tübingen, 72076 Tübingen, Germany; Teresa.Amaral@med.uni-tuebingen.de (T.A.); Claus.Garbe@med.uni-tuebingen.de (C.G.); Heike.Niessner@med.uni-tuebingen.de (H.N.); Tobias.Sinnberg@med.uni-tuebingen.de (T.S.); 2 Institute of Medical Genetics and Applied Genomics, University Hospital Tübingen, 72076 Tübingen, Germany; Franz.hilke@charite.de (F.-J.H.); Axel.Gschwind@med.uni-tuebingen.de (A.G.); German.Demidov@med.uni-tuebingen.de (G.D.); Stephan.Ossowski@med.uni-tuebingen.de (S.O.); Olaf.Riess@med.uni-tuebingen.de (O.R.); Christopher.Schroeder@med.uni-tuebingen.de (C.S.); 3 Institute of Pathology and Neuropathology, University Hospital Tübingen, 72076 Tübingen, Germany; Irina.Bonzheim@med.uni-tuebingen.de (I.B.); Irene.Gonzalez-Menendez@med.uni-tuebingen.de (I.G.-M.); 4 Portuguese Air Force Health Care Direction, 1649-020 Lisbon, Portugal; 5 German DFG NGS Competence Center, NCCT, 72076 Tübingen, Germany; 6 Institute for Clinical Epidemiology and applied Biostatistics (IKEaB), 72076 Tuebingen, Germany; Peter.Martus@med.uni-tuebingen.de; 7 Institute for Radiology, Rems-Murr-Kliniken, 71364 Winnenden, Germany; Bernhard.klumpp@kabelbw.de

**Keywords:** hyperprogression, immune checkpoint inhibitors, acral melanoma, mucosal melanoma, *MDM2*, *MDM4*, *EGFR*

## Abstract

Background: Mucosal and acral melanoma respond worse to immune checkpoint inhibitors (ICI) than cutaneous melanoma. *MDM2/4* as well as *EGFR* amplifications are supposed to be associated with hyperprogression on ICI in diverse cancers. We therefore investigated the response of metastatic acral and mucosal melanoma to ICI in regard to *MDM2/4* or *EGFR* amplifications and melanoma type. Methods: We conducted a query of our melanoma registry, looking for patients with metastatic acral or mucosal melanoma treated by ICI. Whole exome sequencing, FISH and immunohistochemistry on melanoma tissue could be performed on 45 of the total cohort of 51 patients. Data were correlated with patients’ responses to ICI and survival. Results: 22 out of 51 patients had hyperprogressive disease (an increase in tumor load of >50% at the first staging). Hyperprogression occurred more often in case of *MDM2/4* or *EGFR* amplification or <1% PD-L1 positive tumor cells. Nevertheless, this association was not significant. Interestingly, the anorectal melanoma type and the presence of liver metastases were significantly associated with worse survival. Conclusions: So far, we found no reliable predictive marker for patients who develop hyperprogression on ICI, specifically with regard to *MDM2/4* or *EGFR* amplifications. Nevertheless, patients with anorectal melanoma, liver metastases or melanoma with amplified *MYC* seem to have an increased risk of not benefitting from ICI.

## 1. Introduction

Although the introduction of anti-CTLA-4 and anti-PD-1 antibodies has significantly improved the prognosis of metastatic melanoma, primary therapy resistance is still present in about 40%–50% of patients [1,2,3,4,5,6,7]. Furthermore, up to 60% of melanoma patients treated with combined ipilimumab and nivolumab suffer severe, potentially life-threatening immune-related adverse events [8,9,10,11]. Therefore, there is a great need to determine parameters that make response to immunotherapy more predictable. Expression of Programmed cell death ligand 1 (PD-L1) on the tumor cell surface did not prove to be a reliable predictive biomarker for either response or survival, as checkpoint inhibitors are also efficient in PD-L1 negative tumors [12,13,14].

In addition, PD-L1 expression differed in about 50% of cases between primary tumors and metastases, or even between different metastases from one patient [15].

Patients with mucosal and acral melanoma respond worse to immunotherapy than patients with cutaneous melanoma [16,17]. The response rate to anti-PD-1 therapy in patients with mucosal melanoma is only about 23%, and is approximately 37% to combined immunotherapy [16]. Acral melanoma patients also show a reduced objective response rate to anti-PD-1 therapy of around 32% [17].

The situation of mucosal and acral melanoma patients is further complicated by the fact that these tumors are often triple wild-type tumors, i.e., without a *BRAFV600E/K*, *NRAS* or *NF1* mutation, and therefore not qualified for targeted therapy with *BRAF* and MEK inhibitors. On the other hand, triple wild-type melanoma was found to exhibit *MDM2/4* amplifications in about 15% of the cases [18,19]. *MDM2/4* as well as *EGFR* amplifications, have recently been described in association with hyperprogression on ICI in diverse cancers [20].

The term “hyperprogression” describes a fast and extensive progression following treatment with checkpoint inhibitors, but there is no precise and generally agreed definition. A common consensus is most likely to be an acceleration of the tumor growth rate by a factor ≥2 or an increase in tumor burden by more than 50% [21,22,23,24]. Others also considered times to treatment failure of less than two months after initiation of ICI [25]. Not all of these criteria were always met, such as when authors dealt with the term “hyperprogression”. In some case series, staging intervals of three months and more were also included and progression speed was not always calculable [25,26].

We have recently reported a case of an acral melanoma patient with extensive *MDM2* amplification, suffering hyperprogression under combined checkpoint inhibition with ipilimumab and nivolumab. This was probably the first case of *MDM2* amplificated, hyperprogressive melanoma. [27]. Later, an anorectal melanoma patient with hyperprogressive disease under anti-PD-1 therapy was reported [26]. In this second case, however, no information was provided on *MDM2*/*4* or *EGFR* amplification.

In this study, we sought to evaluate the genomic pattern of mucosal and acral melanoma in relation to their response to checkpoint inhibitors. In particular, we intended to check whether *MDM2/4* or *EGFR* amplifications are associated with hyperprogression.

## 2. Materials and Methods

### 2.1. Patients and Clinical Data

**Figure 1:**
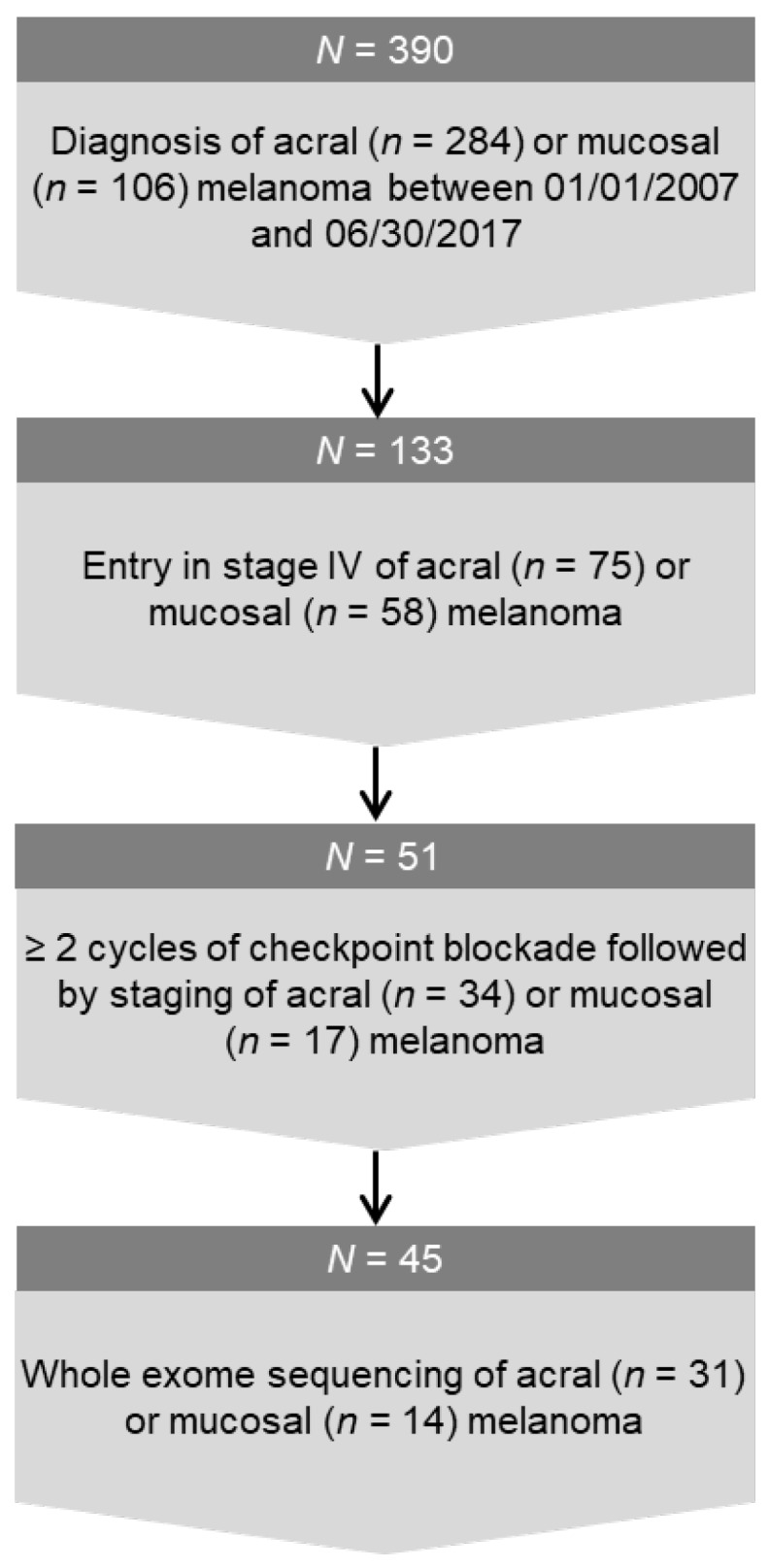
Flowchart of cohort generating.

We conducted a query of our melanoma registry and searched for patients with initial diagnosis of acral or mucosal melanoma in the period 01/01/2007 to 06/30/2017. All patients had given their written informed consent for data collection within the melanoma registry. Among all acral or mucosal melanoma patients identified in the query, all stage IV patients—at the time of first diagnosis or later on—were included for further evaluation if they had received at least two cycles of ICI and a radiological evaluation of response, i.e., CT, MRI or PET/CT scans (Figure 1). The ICI therapy regimes were as follows: ipilimumab (3 mg/kg) every 3 weeks, nivolumab (3 mg/kg) every 2 weeks, pembrolizumab (2 mg/kg) every 3 weeks or combined ipilimumab (3 mg/kg) every 3 weeks and nivolumab (1 mg/kg) every 3 weeks. Two patients had received combined immunotherapy in the frame of a study. The regimes for these two patients were either ipilimumab 3 mg/kg bw and nivolumab 1 mg/kg bw every 3 weeks or ipilimumab 1 mg/kg bw and nivolumab 3 mg/kg bw every 3 weeks. Therapy response was assessed at the first staging through a comparison to baseline evaluation before initiation of ICI. Baseline tumor load and response to therapy were assessed as the sum of long axis diameters of target lesions according to RECIST 1.1. [28]. In this study, complete or partial remission and stable disease (SD) were summarized to the disease control (DC) group. Progression was further classified, either as PD in the case of an increase of tumor load exceeding 20% but limited to 50% and as hyperprogressive disease (HPD) when the tumor burden increased by more than 50%.

Afterwards, the patients’ responses to ICI were classified into 3 categories according to the percentage change in their tumor load:

(1) increase of tumor load of more than 50%: “hyperprogressive disease” (HPD),

(2) increase of tumor load exceeding 20% but limited to 50% “progressive disease” (PD) and (3) up to 20% increase or any decrease of tumor load: “disease control” (DC).

In 4 of the included patients, no CT score could be determined: Two patients had inoperable in-transit metastases, which were not located in the examination area of the CT. Therefore, these two patients were monitored additionally with photos and ultrasound. Both showed progressive findings and were classified as PD due to their clinical and sonographic change in tumor load. Two other patients had their staging outside our institution. Here, the radiology reports were used for response evaluation: one patient had DC and one had PD.

Local ethics committee approved this study (approval number 383/2017BO2). This study was performed in accordance with the Declaration of Helsinki.

### 2.2. Tumor Panel Analysis and Bioinformatics

#### 2.2.1. Whole Exome Sequencing

Whole exome sequencing was performed on an Illumina NovaSeq6000 device (Illumina, San Diego, CA, USA). The library construction was done using the IDT xGen^®^ Exome Research Panel v1.0 (Integrated DNA Technologies, Inc., Carolville, IA, USA) according to the manufacturer’s recommendations by pooling the DNA in batches of 8 samples for library capture. Due to the inadequate DNA integrity number (DIN), we had to adjust the DNA input for each sample. Instead of using 200 ng, we used the total available DNA for each sample. So, we ended up generating 11 pools with a starting amount ranging from 510–39 ng. The resulting 8 sample library pools were paired-end sequenced.

Whole exome sequencing quality control: An in-house developed pipline, called “megSAP” was used for data analysis (https://github.com/imgag/megSAP, vers. 0.1-484-g9ad29f4 and 0.1-614-g21d6cfe). In brief, sequencing reads were aligned to the human genome reference sequence (GRCh37) using BWA (vers. 0.7.15) [29]. Quality control parameters, like sample or data swaps as well as all meta data were collected during all analysis steps [30].

#### 2.2.2. Somatic Mutation Analysis

Variants were called using Strelka2 (vers. 2.7.1) [31] and annotated with SNPeff/SnpSift (vers. 4.3i) [32]. For further interpretation, all somatic variants were uploaded to the Cancer Genome Interpreter [33].

#### 2.2.3. Tumor Mutational Burden

The tumor mutational burden (TMB) was calculated as the number of all somatic alterations (coding SNVs and INDELs) based on the target size of the used exome enrichment system.

The formula was:

$$\left[\frac{\left(\frac{Somatic-Known-Tumorgenes}{Target\;size}\times Genome\;size\right)+Tumorgenes}{Genome\;size}\right].$$

#### 2.2.4. Copy Number Analysis

Copy number variants (CNVs) were identified using ClinCNV [34], an algorithm for multi-sample CNVs detection using targeted or whole-genome NGS data. Visual inspection of CNVs was performed. Samples were filtered out in case of a noisy coverage plot, large number of short homozygous deletions or complete absence of CNVs. Thus, we ended up with the calls of 40 patients. Ploidy was calculated as an average copy-number of the genomic regions within the sample. All CNVs calls were corrected for purity, cellularity and ploidy. Point mutations with the predicted loss-of-function effect were analyzed together with deletions. All detected CNVs were uploaded to CancerGenomeInterpreter.org for further interpretation. Copy numbers ≥4 were defined as amplifications in line with previous publications by Hayward et al. [18] and Liang et al. [35]. Copy numbers <1 were defined as deletions.

### 2.3. Immunochistochemistry and Fluorescence In-Situ Hybridization

#### 2.3.1. Immunohistochemistry

Immunohistochemistry (IHC) was performed on formalin-fixed, paraffin-embedded (FFPE) tissue sections on the Ventana Ultra automated staining System (Ventana Medical Systems, Oro Valley, AZ, USA) using Ventana reagents, according to the manufacturer´s protocol. All cases were stained with an antibody against PD-L1 (clone 22C3, order no. M3653, 1:50, Dako, Cambridge, UK), MDM2 (clone 3G187, order no. 113-0230, 1:25, Zytomed, Berlin, Germany). Primary antibody detection was performed using the OptiView DAB IHC detection kit (Ventana). Appropriate positive and negative controls were used to confirm the adequacy of the staining. PD-L1 expression was evaluated using the MEL Score (PD-L1 positive tumor cells + PD-L1 positive mononuclear inflammatory cells/total tumor cells + total mononuclear cells). We classified PD-L1 staining into two groups: MEL score <1 and ≥1. MDM2 expression was specified as percentages of positive tumor cells. Images were acquired with a Zeiss Axioskop 2 plus microscope equipped with a Jenoptik ProgRes C10 plus camera and software (Laser Optik System, Jena, Germany).

#### 2.3.2. Fluorescence In-Situ Hybridization Analysis of MDM2

Fluorescence in-situ hybridization (FISH) analysis of *MDM2* gene amplification was performed using ZytoLight^®^ SPEC *MDM2*/CEN 12 Dual Color Probe (order no. Z-2013, ZytoVision, Bremerhafen, Germany). Pretreatment and hybridization (HYbrite™, Abott, Wiesbaden, Germany) was done according to the manufacturer’s manual. Assessment of results was carried out on the Axio Imager M2 (Zeiss, Oberkochen, Germany).

### 2.4. Statistical Analysis

Statistical analysis was performed using the statistical program for social sciences (SPSS) Version 25 (IBM, New York, NY, United States). STATA^®^ v15 (StataCorp LLC, College Station, TX, USA) was used to generate the final version of the Kaplan-Meier survival curves. Differences between groups were tested using the Exact Fisher test and the exact version of the Chi-Square test for categorical variables (response and comparisons between potential predictors), and the Log rank test for overall survival. Survival curves were generated according to the Kaplan-Meier method. Overall survival was defined as the time between start of immunotherapy and death or censored at the last date of patient contact. For factors that were significant in the univariate log rank test, we performed a multivariate Cox regressions analysis. In this analysis, the three categorical variables “melanoma subtype” and “treatment response” were coded quantitatively as 1 = other mucosal melanoma, 2 = acral melanoma, 3 = anorectal melanoma (melanoma subtype), and 1 = disease control, 2 = progressive disease, 3 = hyperprogressive disease (treatment response). The level of significance was 0.05 (two-sided) in all analyses. In an ex post power analysis, we found that hazard ratios of 2.7 or higher and differences in frequencies of 42% could have been detected with 80% power in our sample (assuming equal group sizes).

## 3. Results

### 3.1. Patient Cohort and Clinical Parameters

The query of the melanoma registry identified 390 patients who had been diagnosed with acral (*n* = 284) or mucosal (*n* = 106) melanoma in the time between 01/01/2007 and 06/30/2017. About one third of the cohort (*n* = 133, 34%) entered stage IV during the course of the disease and 51 of these patients received at least 2 cycles of checkpoint inhibitors for unresectable metastases (Figure 1). Of these 51 cases, *n* = 34 were acral and *n* = 17 were mucosal melanoma.

The median age of the patients at the time of initiation of ICI was 71 years (IQR 58–77, range 40–87). About half of the patients were female (47%). Among the 17 mucosal melanomas, 8 were anorectal. About half of the patients (*n* = 27) had been treated by ipilimumab, 15 patients by anti-PD-1 therapy and 9 by a combination of ipilimumab and nivolumab. The median time between the start of immunotherapy and the first staging was 11 weeks (IQR 8–13).

The results of the first staging after starting the ICI were PD in the majority of the 51 patients (67%). Patients with anorectal melanoma responded particularly poorly: Of the eight patients with anorectal melanoma, seven had PD (88%). The patients with mucosal melanoma had a better response to therapy. Here only 33% had PD (Table 1). Six out of 8 patients (75%) with anorectal melanoma developed hyperprogression compared to 16 out of 43 patients (37%) with acral or other forms of mucosal melanoma.

A total of 37 out of 51 patients (73%) had already died at the time of the evaluation. Median overall survival of the total 51 patients since primary diagnosis of melanoma was 40 months, and the equivalent since the start of immunotherapy was 19 months. Anorectal melanoma patients had the worst prognosis with a median overall survival of 16 months and 8.5 months, respectively.

### 3.2. The Genomic Landscape of the Patients’ Cohort

Of the final cohort of 51 cases, 45 patients had formalin-fixed tissue available for WES and IHC staining. For 6 patients, no tissue was accessible (Figure 1).

Paired tumor normal exome sequencing was performed with an average depth of 154× (range: 10.36–440.1) for the tumor tissue and 170× (range: 15.91–467.6) for normal tissue. We were able to call somatic single nucleotide variants (SNVs) and small insertions and deletion (INDELs) in all cases, but had to exclude five samples from the detection of the somatic copy number analysis due to poor tissue quality.

**Figure 2:**
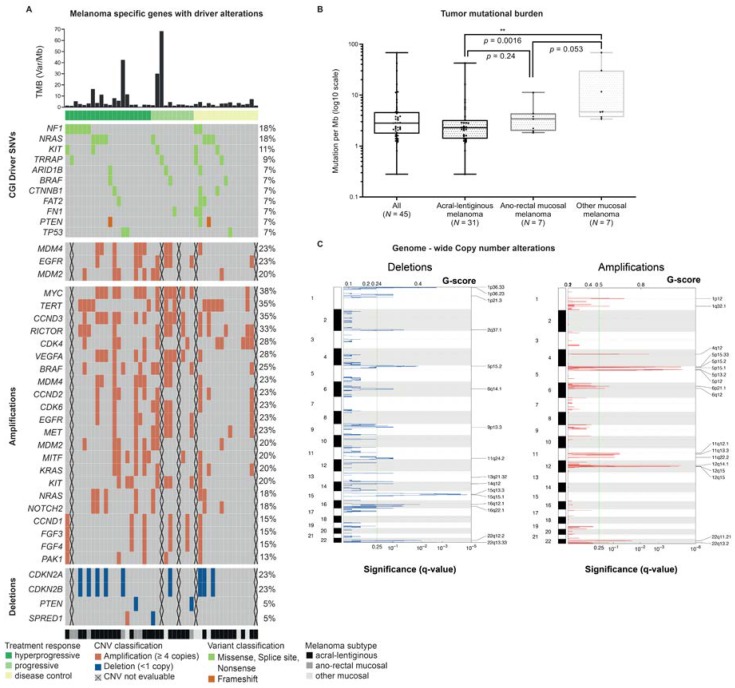
Mutational landscape of the cohort: tumor mutational burden (TMB), single nucleotide variants (SNVs), copy number variants (CNVs). The figure shows the most frequent alterations found in the 45 cases analyzed by whole-exome sequencing. The oncoplot (**A**) Melanoma specific genes with driver alterations depicts a list of genes filtered with the *Cancer Genome Interpreter* for known and predicted driver alterations. The upper part shows the tumor mutational burden (TMB) of each patient shown on a decimal scale (Mut/Mb). The oncoplot presents in the second panel from the top all genes with at least 3 mutations (SNVs and INDELs) in two different patients, translating to a minimal alteration frequency of 7%. The genes are sorted according to their alteration frequency and alphabetically. Each row represents a patient. The patients are sorted according to their melanoma subtype (acral-lentiginous, anorectal mucosal and other mucosal melanoma). Also, the response to the checkpoint inhibitors is shown (hyperprogression, progressive disease and disease control). In addition, the two lower panels depict somatic copy number alterations of particular interest. The lower panel shows somatic SNV and alteration frequency of all driver alterations found in the significantly altered genomic region shown in (**C**). The middle panel displays *MDM4*, *EGFR* and *MDM2*. On the right hand (**B**), the upper part Tumor mutational burden depicts the results of the comparison of the TMB between the three different melanoma subtypes. The boxplots show all data points, median, interquartile borders as well as minimum and maximum. The *t*-test (Wilcoxon-rank test) revealed a significant difference while comparing the TMB of acral-lentiginous (median: 2.28, range: 0.28–42.54) and other mucosal melanoma (median: 4.68, range: 3.38–68.34), meaning in acral-lentiginous melanoma, a significant lower TMB was observed compared to other mucosal melanoma. ** significant difference in the Wilcoxon-rank test. The lower part (**C**), Genome–wide Copy Number Alterations presents the results of the GISTIC analysis. GISTIC identified 16 regions on 11 chromosomes to be significantly deleted and 18 regions on 7 chromosomes to be significantly amplified. The cut-off for calling the regions is 0.25 (q-value).

In total, we identified 8297 somatic SNVs. The analysis of the coding variants resulted in a median tumor mutation load of 2.83 mutations/Mb (range: 0.28–68.34). The tumor suppressor gene *NF1* and the oncogene *NRAS* were the most frequently mutated genes, each of them occurred in 18% (8 of 45) of the cases. The second most common gene concerned the oncogene *KIT* (5 of 45, 11%) (Figure 2A). We found driver mutations in the oncogene *BRAF* exclusively in patients with acral melanoma.

Out of all 45 samples in which tumor mutation burden (TMB) determination was possible, 60% were classified as TMB low (<3.3 Mut/Mb), and about a third as intermediate (3.3–23.1 Mut/Mb). Only 3 patients (7%) had a high TMB (>23.1 Mut/Mb) [3].

The median TMB of the total cohort was 2.8 Mut/Mb (IQR 1.75–4.65). There was no significant difference in median TMB of patients with DC (2.4; IQR 1.3–4.6) and PD (2.8 Mut/Mb; IQR 2–5.4). The total cohort contained only three patients with a high TMB: one patient with acral melanoma who presented with PD and two patients with other types of mucosal melanoma and DC, respectively. There was no statistically significant difference between the three TMB groups and treatment response, classified in HPD, PD and DC. Interestingly, non-anorectal mucosal melanomas had a significantly higher TMB than anorectal or acral melanomas (Figure 2B).

Evaluation of copy number changes using GISTIC 2.0 showed that 18 regions were frequently amplified and 16 regions were frequently deleted (q < 0.25) (Figure 2C). The most frequently significantly (q < 0.05) amplified regions were located on chromosome 5p33.1, 12q14.1/15, 4q12 and 1p12 comprising the genes *TERT*, *CDK4*, *MDM2*, *KIT* and *NRAS*. The most frequently significantly deleted regions were located on chromosomes 15q15, 22q12/13, 1p36, 16q12/22, 5p15 and 2q37 affecting the tumor suppressor genes *SPRED1*, *CHEK2* and *NF2* (Figure 2C).

We furthermore evaluated other oncogenes or tumor suppressor genes, known to be frequently amplified or deleted in acral or mucosal melanoma [35,36] and performed a comparison with the Cancer Genome Interpreter database. Oncogenes with amplifications (copy number ≥4) and tumor suppressor genes with deletions (copy number <1), respectively are presented in Figure 2A. The five most common oncogenes concerned *MYC*, *TERT*, *CCND3*, *RICTOR* and *CDK4*. The most commonly affected tumor suppressor genes involved *CDKN2A/B*.

### 3.3. MDM2 FISH and PD-L1 Immunohistochemistry

**Figure 3:**
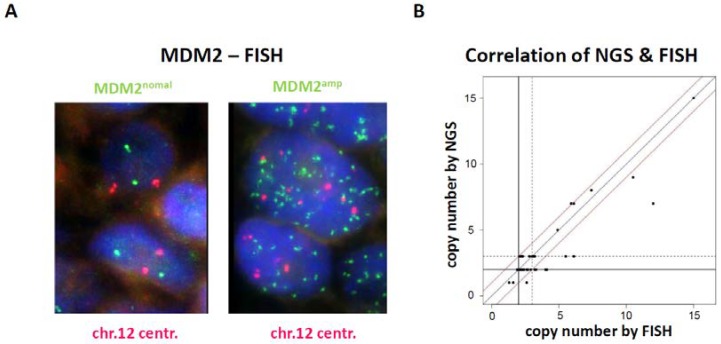
*MDM2* fluorescence in-situ hybridization (FISH). (**A**) Left microphotograph shows a representative case without *MDM2* amplification (*MDM2*^normal^). On average, 2.7 copies of *MDM2* per tumor cell nucleus were detected by green fluorescence signals and 2.2 copies of centromer 12 per nucleus were detected by red fluorescence signals (*MDM2*/centromere 12 ration: 1.22). Right microphotograph shows a representative case with *MDM2* amplification (*MDM2*^amp^). (**B**) Correlation diagram of *MDM2* copy numbers obtained by FISH and NGS.

FISH was performed using a *MDM2* specific probe to confirm the NGS results for copy number changes in *MDM2*. In the absence of a *MDM2* amplification, two positive signals are to be expected in both: the 12q15 (green) signals and the centromere (red) signals by FISH. An example of this is given in Figure 3A (left side). In contrast, numerous signals can be detected in the case of *MDM2* amplifications, as shown in Figure 3A (right side). Remarkably, the FISH results correlated very well with the results of the NGS study (Figure 3B). However, immunohistochemistry (IHC) for MDM2 did not correlate very well with the copy numbers determined by NGS or FISH (Pearson *r* = 0.46). This might be explained by a strong regulation of MDM2 expression e.g., by varying p53 activity or post-translational regulation in the tumor cells. Furthermore, no scoring of the staining intensity was used for the quantification of IHC. Immunohistochemistry for detection of PD-L1 expression was performed in all melanoma samples and further used to determine the PD-L1 score. Results of PD-L1 staining ranged from 0% to 40% of PD-L1 positive cells (IQR 0.875–6.25), Table S1 displays PD-L1 scores in detail. Furthermore, Figure S1 shows PD-L1 IHC and MDM2 IHC of a hyperprogressive case, and of a case with disease control.

### 3.4. Response to Immune Checkpoint Inhibitors-Anorectal Melanomas are Associated with Hyperprogressive Disease

Patients with the anorectal melanoma type often had significantly more hyperprogressive courses than the other two melanoma subtypes. Hyperprogression was observed in 75% of all anorectal melanoma patients, but only in 41% of the acral and in 22% of the other mucosal melanomas (*p* = 0.012).

About 90% of all patients whose tumors harbored amplification of *MDM2* (7 out of 8), *MDM4* (8 out of 9) or *EGFR* (8 out of 9) developed PD and 66% of the patients with *MDM4* amplification presented with HPD (6 out of 9).

Although we did not find a statistically significant difference, there was a trend towards a worse response in case of amplification (≥4 copies) of *MDM2*, *MDM4* or *EGFR* or PD-L1 score <1%. (Table 2). It should be pointed out that we have not obtained different results, even when combining the two groups HPD and PD to one single group of progressive patients (Table S2). Again, we only found a statistically significant difference when analyzing the melanoma subtype. Note that patients’ age had no influence on treatment response (*p* = 0.40). However, *MYC* copy numbers ≥4 were significantly associated with PD (*p* = 0.013).

### 3.5. Survival Analysis-Anorectal Localization and Presence of Liver Metastases are Independent Negative Influencing Factors on Survival

**Figure 4:**
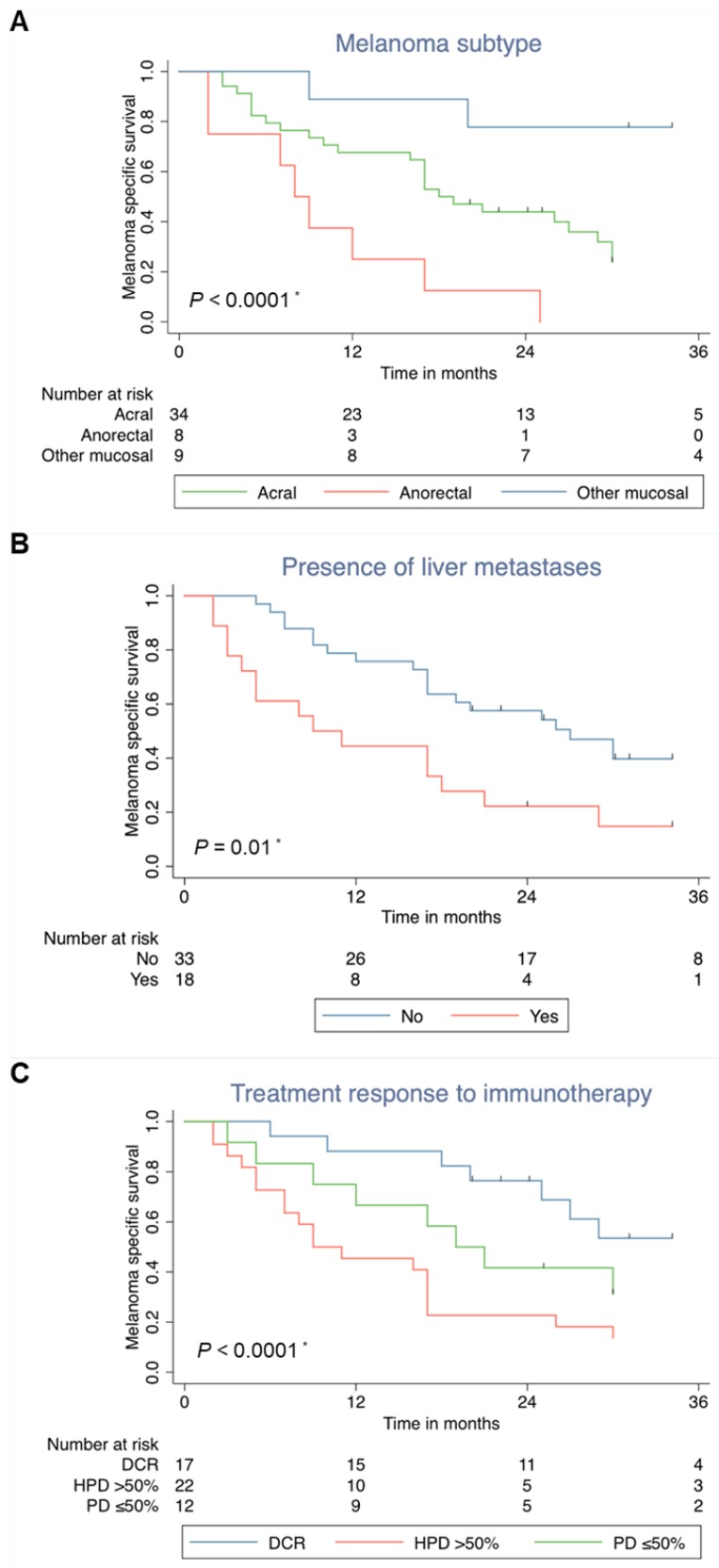
Melanoma specific survival according to (**A**) melanoma subtype (acral, anorectal, other mucosal melanoma), (**B**) liver metastases (present, absent) (**C**) response to checkpoint inhibitor (disease control, progressive disease, hyper-progressive disease). * significant difference in the Log rank test.

The univariate survival analysis revealed significant negative influence factors for survival: the anorectal melanoma subtype, presence of liver metastases and HPD in the first staging (Figure 4). Patients with amplification (≥4 copies) of *MDM2, MDM4* or *EGFR* had no worse survival than patients without these amplifications (Figure S2). Note that patients’ age was no influence factor for survival (HR = 0.99, CI 0.96–1.01).

The multivariate Cox regressions analysis revealed that liver metastases at start of ICI ((HR) 2.07; 95% confidence interval (CI) 1.05–4.08)), and the anorectal melanoma subtype ((HR 7.92; 95% CI 2.22–28.38)) are independent negative influencing factors on survival. Even after inclusion of the variable “treatment response”, which is an intermediate variable (HR = 1.91, CI 1.24–2.93), both prognostic factors remained significant (liver metastasis HR = 2.44, CI 1.18–5.01, anorectal melanoma subtype HR = 2.13, CI 1.09–4.17).

## 4. Discussion

The aim of this study was to check possible correlations between hyperprogression to checkpoint inhibitors and amplification (≥4 copies) of *MDM2*, *MDM4* or *EGFR* in acral and mucosal melanoma. We furthermore tested PD-L1 expression and clinical parameters such as liver metastases and specific melanoma subtypes and obtained relevant results. Although we could not see a statistically significant association between amplification of *MDM2*, *MDM4* or *EGFR* and the response to checkpoint inhibitors, we found a trend towards a lower chance of disease control in case of either *MDM2, MDM4* or *EGFR* amplification. The anorectal melanoma subtype was associated with both significantly increased risk of having HPD and worse survival. Another factor for poor survival, which was independent of the anorectal type of melanoma, was the presence of liver metastases at the time of the start of ICI.

The influence of *MDM2/4* and *EGFR* amplification on response to immunotherapy is not completely clarified. *MDM2* and *MDM4* (an homologue of *MDM2*) inhibit the p53 tumor suppressor [37]. Likewise, it is known that *EGFR* activation might be associated with an upregulation of the checkpoints CTLA-1, PD-1 and PD-L1, which in turn, can cause resistance to checkpoint inhibitors [38]. Thus, *MDM2/4* and *EGFR* amplification have been brought into context with the development of hyperprogressive disease but the underlying mechanisms are not fully understood [20,27]. We found a trend but no significant correlation between worse response to checkpoint inhibition and either *MDM2, MDM4* or *EGFR* amplification, what is probably due to the relatively small number of cases. Furthermore, it has to be noted that only extremely effects (hazard ratios of 2.7 and differences of frequencies of 42%) could have been detected with our sample. Thus, negative results in our analysis do not confirm absence of true effects. In addition, it must be stated that we also had three different patients with disease control, despite the presence of *MDM2*, *MDM4* or *EGFR* amplification. Therefore, at the current state of knowledge, the presence of one of these amplifications should not be regarded as an exclusion criterion for checkpoint inhibitor therapy.

The NGS and FISH results showed a high degree of agreement, which confirmed the reliability of these two methods. The overall low TMB of our collective with a median of only 2.8 Mut/Mb fits well to other studies, according to which mucosal and acral melanoma are tumors with significant lower TMB than cutaneous melanoma [18,39]. Given the lower TMB of mucosal and acral melanoma compared to cutaneous melanoma, it might not be surprising that the response to checkpoint inhibition in these melanoma subtypes is lower. Retrospective analyses revealed 23% to 37% overall response rates for mucosal or acral melanoma patients treated by immunotherapy [16,17,39,40].

In a large study of 102,878 patients with different malignancies, NGS of tumor tissue revealed *MDM2* amplification in 3.5% of cases [41]. Tumors exhibiting *MDM2* amplification were less often associated with a high TMB than *MDM2* wild-type tumors [41]. We cannot validate this observation in our cohort, as only three patients had a high TMB and the median TMB in our cohort was very low. Nevertheless, there was no significant difference in the median TMB between patients with DC (2.4 Mut/Mb) and patients with PD (2.8 Mut/Mb).

It is noteworthy that most of the patients (75%) with anorectal melanoma developed hyperprogressive disease. We have also seen that PD-L1 expression was significantly lower in this subtype than in the others. Furthermore, it was shown by others, that anorectal melanoma is associated with low TMB and a low number of tumor infiltrating lymphocytes. Therefore, these tumors are probably less immunogenic and thus less responsive to checkpoint inhibitors [39]. Whether the hyperprogression was triggered by immunotherapy or simply by the natural course of the disease cannot be clarified.

In our cohort, patients with liver metastases had a significant worse rate of survival. The inferior outcome of patients with liver metastases treated by ICI has already been shown in other collectives, including patients treated with pembrolizumab and combined ipilimumab and nivolumab [42,43,44]. This might be due to a lower density of CD8^+^ T cells in the liver metastases and also in the distant non-liver metastases of these patients [42]. In this context, it should be mentioned that an immunotolerance inducing effect resulting from the liver tissue is well-known in the field of liver transplantation. Even in the absence of histocompatibility, immunosuppression is not always required in case of transplanted liver tissue [45]. Furthermore, if several organs of one single donor are transplanted, the probability of successful transplantation increases if the liver is also transplanted [46]. This liver-induced immunotolerance might be disadvantageous in view of checkpoint inhibitors. In case of *BRAF V600* positive melanoma, treatment with BRAF and MEK inhibitors might be more suitable in case of liver metastases, as we did not find any reports indicating a worse response of liver metastases to targeted therapy.

Our study has several strengths. The systematic and wide-reaching query of our melanoma register provides an important basis, as all mucosal and acral melanomas that had received at least 2 cycles of checkpoint inhibitors could be identified and recorded in this study. Cases with only one cycle were omitted, because from our point of view in such cases the effect of the immunotherapy cannot be evaluated sufficiently. In our opinion, a representative collective was assembled and reported here.

Our study also presents some limitations. It should be noted that we could not exactly screen out patients for hyperprogression as defined by Champiat and colleagues [25] due to the retrospective character of our study. Response to therapy is normally performed every 12 weeks in as part of a daily routine. Therefore, we could not find a time to treatment failure of less than 2 months with simultaneous doubling of the growth rate in this time. Nevertheless, we were able to identify hyperprogressive patients by a >50 % increase in tumor mass in the first staging.

Next, it should be mentioned that only 18% of our patients were treated with the combination of ipilimumab and nivolumab. This is due to the systematic and far-reaching survey of cases with primary diagnosis between 2007 and 2017, which was necessary to generate a sufficiently large number of cases due to the rarity of these melanoma subtypes. The combination of ipilimumab and nivolumab was not approved in Germany until 2016, whereas ipilimumab had been approved since 2011. Since the combination of ipilimumab and nivolumab is superior to monotherapy with ipilimumab or nivolumab [10], it should be examined whether the worse response in the case of *MDM2*, *MDM4* or *EGFR* amplification also occurs in patients treated with combined immunotherapy.

Our finding that MYC amplifications are associated with ICI therapy failure suggests a potential biomarker role of MYC for primary resistance to ICI therapies. This is in line with recent experimental findings by others, who mentioned that MYC family members regulate the gene expression of immune checkpoints, including PD-L1 and CD47 [47].

Finally, the limited sample size of the study does not allow us to draw definite conclusions from negative results.

## 5. Conclusions

Patients with acral or mucosal melanoma harbor a high risk of not responding to checkpoint inhibitors and should be monitored closely. Patients with anorectal melanoma, melanoma with amplified *MYC* or liver metastases have an additional risk of not benefiting from checkpoint inhibitors and might have a significantly worse survival. In addition, in this collective, patients with *MDM2*, *MDM4* or *EGFR* amplification have an increased risk of not responding to checkpoint inhibitors. Despite this, a few patients with such amplifications did develop disease control. Therefore, these patients should not be excluded from immunotherapy in general. In these cases, the chance of response should be carefully weighed against possible risks, such as severe side effects, especially if the patients’ performance status is reduced.

Further studies, including NGS in a sufficient lager number of patients with liver metastases and anorectal melanoma should be carried out to investigate the phenomenon of hyperprogression in melanoma.

## Figures and Tables

**Table 1:** Patient characteristics.

Patient Characteristics (*n* = 51)		Median	IQR
Age at first diagnosis of melanoma (years)		72	60–79
Tumor mutation burden (Mut/Mb)		2.8	1.8–4.7
Overall survival since primary diagnosis (months)		40	23–78
Overall survival since start of immunotherapy (months)		19	9–30
Time between tissue sampling and start of combined immunotherapy (months)		4	1–12
Time between start of combined immunotherapy and first staging (weeks)		11	8–13
		no. patients	%
Sex			
	Female	24	47
	Male	27	53
Melanoma type			
	Anorectal	8	15.7
	Acral	34	66.7
	Other mucosal	9	17.6
LDH at start of immunotherapy			
	LDH elevated	17	33.3
	LDH normal	32	62.7
	missing	2	4
Metastasis at start of Immunotherapy			
	Brain metastasis	6	11.8
	Liver metastasis	18	35.3
	Lung metastasis	31	60.8
Type of immunotherapy			
	Ipilimumab	27	53
	Nivolumab or Pembrolizumab	15	29.4
	Combined Ipilimumab + Nivolumab	9	17.6
Response to immunotherapy			
	Disease control	17	33.3
	Progressive disease	12	23.5
	Hyperprogressive disease	22	43.1
Origin of tissue sequenced			
	Metastasis	35	68.6
	Primary melanoma	10	19.6
	No tissue available	6	11.8

**Table 2:** Characteristics and ICI treatment response. ^a^ Genetic pattern and clinical characteristics according to treatment response. ^b^ Genetic pattern and clinical characteristics according to melanoma subtype.

Category ^a^	Total *n* = 51	Hyperprogressive Disease *n* = 22	Progressive Disease *n* = 12	Disease Control *n* = 17	*P*-value ^1^
MDM2 Amplification (≥4 copies) ^2^					0.273
Present	8	5	2	1	
Absent	32	14	6	12	
MDM4 Amplification (≥4 copies) ^2^					0.142
Present	9	6	2	1	
Absent	31	13	6	12	
EGFR Amplification (≥4 copies) ^2^					0.534
Present	9	4	4	1	
Absent	31	15	4	12	
PD-L1 Score ^3^					0.190
<1%	11	7	2	2	
≥1%	35	15	6	14	
Melanoma Type					0.012 *
Other mucosal	9	2	1	6	
Acral	34	14	10	10	
Anorectal	8	6	1	1	
Liver metastases					0.319
Present	18	10	3	5	
Absent	33	12	9	12	
Age (median 71 years)					0.397
<71 years	24	8	3	13	
≥71 years	27	9	9	9	
**Category** **^b^**	**Total** ***n* = 51**	**Anorectal Melanoma** ***n* = 8**	**Acral Melanoma** ***n* = 34**	**Other Mucosal Melanoma** ***n* = 9**	***P*-value** **^1^**
MDM2 Amplification (≥4 copies) ^2^					0.721
Present	8	1	7	0	
Absent	32	5	21	6	
MDM4 Amplification (≥4 copies) ^2^					1.0
Present	9	2	5	2	
Absent	31	4	23	4	
EGFR Amplification (≥4 copies) ^2^					0.303
Present	9	3	5	1	
Absent	31	3	23	5	
PD-L1 Score ^3^					0.004 *
<1%	11	5	6	0	
≥1%	35	3	23	9	
Liver metastases					0.316
Present	18	4	12	2	
Absent	33	4	22	7	
Age (median 71 years)					0.799
<71 years	24	4	15	5	
≥71 years	27	4	19	4	

* Significant. ^1^ Exact Chi-Square Test for Trend (Monte Carlo Simulation). ^2^ only cases where tissue was available and CNV were evaluable could be considered, therefore 11 cases are missing. ^3^ only cases where tissue was available could be considered, therefore 6 cases are missing.

## Supplementary Materials

The following are available online at https://www.mdpi.com/2072-6694/12/3/540/s1, Table S1: PD-L1 scores of the cohort, Figure S1: MDM2 and PD-L1 immunostaining in a hyperprogressive case (39T) and in a case with disease control (30T) (original magnification, ×200), Table S2: Genetic pattern and clinical characteristics according to treatment response, Figure S2: Melanoma specific survival according to (A) MDM2 amplification (≥4 copies vs. <4 copies), (B) MDM4 amplification (≥4 copies vs. <4 copies) (C) EGFR amplification (≥4 copies vs. <4 copies), (D) PD-L1 scores (≥1% vs. <1% positive tumor cells).
